# School refusal: a hidden challenge for working parents

**DOI:** 10.1093/joccuh/uiaf039

**Published:** 2025-07-09

**Authors:** Natsu Sasaki

**Affiliations:** Department of Mental Health, Graduate School of Medicine, The University of Tokyo, Bunkyo-ku, Tokyo, Japan

**Keywords:** parents, work–family, mother, father, mental health, well-being

## Abstract

School refusal is rising globally and has reached an all-time high in Japan. It imposes critical work–family conflict on employed parents, with preliminary surveys linking it to job loss, depression, and anxiety. Despite its public health importance, work–family conflict due to school refusal has scarcely been studied in occupational health. This Opinion reviews existing research on parents of school-refusing children, discusses how occupational health professionals can support this underserved population, and outlines priorities for future research to mitigate the impacts on working-age parents’ health and employment.

## 1. Need for support for working parents of a child with school refusal

School refusal is increasingly recognized not only as an educational and mental health issue affecting children, but also as a significant source of stress and work–family role conflict for working parents. Some parents’ feelings of helplessness and uncertainty about how to handle their children’s school refusal can negatively affect their employment and mental health. A survey by a Japanese Non-Profit Organization in 2024 revealed that 80% of parents reported that their employment was impacted, and 1 in 4 parents had resigned their employment.^1^ Over 60% of those who had left their jobs cited instability in their own mental health as one of the reasons. Although academic data about the impacts on parents’ employment and work are limited, there is little doubt that such parents constitute a population in urgent need of support.

Recent tragic events in Japan have highlighted the emotional toll faced by caregivers of children who experience school refusal. In December 2024, a working mother in Japan killed her 9-year-old son and her 2 daughters (aged 13 and 15), then attempted suicide. Reports indicated that her son had been experiencing school refusal and she struggled with parenting.^2^ Media reported that she was employed, lived with relatives in the same building, and had access to various support resources. She had sought help from child welfare services 12 times and regularly consulted a school counselor. Yet, no agency was able to intervene in time to help her or save her children.

## 2. School refusal reaches an all-time high in Japan

The most recent national survey conducted in 2023 reported that approximately 350 000 elementary and junior-high students in Japan had chronic absenteeism, the highest number on record.^3^ Since the COVID-19 pandemic, the phenomenon of school refusal has increased worldwide. In the United States particularly, chronic absenteeism has spiked dramatically—with an additional 6.5 million students affected due to the pandemic.^4^ In the United Kingdom, rates of chronic absenteeism have nearly doubled compared with pre-pandemic levels.^5^ In Japan, the increase over the past decade is even more pronounced: school refusal has increased 2.7-fold in elementary schools and 3.5-fold in junior high schools. Currently, 37.2 of every 1000 students in Japan miss more than 30 days of school per year for psychosocial reasons.^3^

## 3. Causes of school refusal

Emotionally based school avoidance (EBSA)—in which children avoid school primarily due to emotional distress—accounts for most school refusal cases. Although EBSA is not an official medical diagnosis, many children who avoid school for emotional reasons have underlying anxiety-related conditions. Previous research has identified a broad range of contributing factors, including child-related factors (eg, special educational needs, health problems), parental factors (eg, family functioning), and environmental factors (eg, school climate, bullying, and student–teacher relationships).^6^ However, the specific reasons and causes are unknown and complicated in many cases. Focusing on identifying the cause may, in some cases, cause additional distress for both the child and the parent, and does not necessarily lead to effective solutions.

## 4. Consequences of school refusal

Parents are vulnerable to the negative effects of school refusal. Compared with parents whose children continue to attend school, parents of children exhibiting school refusal reported higher levels of anxiety, depression, and lower self-efficacy.^7^^,^^8^ Parents are frequently compelled to take emergency leave or adjust their working hours to accommodate their children’s early school departures or absences, resulting in friction with employers and reduced income.^8^ Both financial pressures (the risk of losing work pay) and temporal pressures (eg, making up for lost working time) can adversely affect working parents. Although flexible working arrangements may provide some respite, they often involve financial and temporal trade-offs.^8^

Some parents feel that keeping their child off school is unhelpful in the long run, but they emphasize the difficulty of making an anxious child go to school.^9^ Home is a safe place for children; in fact, avoiding school may sometimes improve children’s mental health and even reduce the potential for suicidality. Parents often face a dilemma: keeping children safe at home or ensuring they go to school, a vital learning environment for children’s social and academic development. Parents often struggle to determine whether returning to school should be their sole goal or not. Besides, they often feel shamed and blamed,^10^ leading to reduced motivation to seek help from others. Consequently, parents can easily become overwhelmed and isolated.

## 5. Solutions for school refusal

A review concluded that early interventions involving parents and cognitive behavioral therapy (CBT) for the child effectively improve school attendance.^6^^,^^11^^,^^12^ The efficacy of oral medication for children remains limited.^11^^,^^12^ Since 2000, scholars have emphasized the need for an integrated approach involving the child, parent, and school in addressing school refusal.^13^ Such multilevel early interventions, combining a CBT-based approach and school-based accommodations, have been recommended as the first line of treatment. However, such interventions remain inaccessible to the vast majority of families. Moreover, approaches that focus on parental mental health have not been implemented yet.

## 6. Potential contributions by employers

Since the 1980s, when the concept of work–family conflict became widely recognized, supporting employees in balancing work and family responsibilities has emerged as a critical issue for employers.^14^ When a child refuses to attend school, working parents are often required to devote substantial time and emotional energy to coordinating with support agencies and managing family-related issues on a daily basis.^13^ This burden extends beyond the private domain, potentially affecting workplace productivity and employee well-being.^15^ In the post-COVID era, it is no longer sufficient for organizations to simply offer flexible working arrangements. A more individualized approach is needed, particularly for employees with caregiving responsibilities.^16^^,^^17^ However, many employees hesitate to tell their employers when their child is not attending school. A lack of awareness—and the invisibility of their burden—can result in a failure to provide adequate accommodations. Therefore, companies must actively cultivate a culture in which employees feel comfortable sharing family issues. Open dialogue between employees and employers enables the identification of appropriate measures and the tailoring of accommodations to meet individual needs.

## 7. What can occupational health professionals and human resources departments do?

To prevent employees’ depression and fatigue, occupational health professionals have to pay attention not only to employees’ work stress but also to their family stresses or burdens.^18^ In this regard, occupational health professionals can:

- Assess employees’ mental health and, if necessary, facilitate referrals for treatment.
- Provide suggestions on mental health care.
- Assess the need to plan feasible job accommodations.
- Gather information to anticipate the health impacts of work–family conflicts.
- Offer medical advice concerning family members, within their scope of expertise.

Together, occupational health professionals and human resources (HR) can raise organizational awareness of hidden family-related challenges (including eldercare), foster an inclusive workplace, establish internal support systems (eg, leave, economic assistance for external service use), and promote workplace–community partnerships to connect employees with local resources.

## 8. Practical messages

This complex, multidisciplinary issue demands urgent attention from public health perspectives—rapid, decisive implementation is essential. The factors contributing to school refusal are diverse, but this heterogeneity should not be an excuse for failing to provide effective interventions. However, it may take a long time to establish community-based or school-based solutions. It is the role of occupational health professionals and HR staff to provide immediate support that addresses the needs of workers who are adversely affected by the growing conflict between work and family responsibilities. The widespread reliance on a “wait-and-see” approach, the effectiveness of which remains uncertain, can sometimes exacerbate working parents’ distress due to its lack of a clear outlook; having supportive advocates within the workplace therefore provides essential assistance. There is an urgent need to develop and implement support measures, such as connecting with community resources and ensuring the availability of relevant internal workplace programs.

## 9. Future research

Empirical research on the mental health of working parents raising school-refusing children is limited. Although several qualitative studies have explored this issue,^9^^,^^19^^-^^21^ there is a critical need for epidemiological research to identify specific support needs and inform policy and intervention development. We have yet to establish parents’ true needs, worries, desires, and values, especially regarding their work. We should explore the issue, integrating with existing and newly proposed occupational health topics, like workplace loneliness,^22^^,^^23^ work–family spillover,^24^ and flexible work. Furthermore, it is essential to develop and evaluate effective measures that occupational health professionals and HR departments can implement to alleviate this distinctive form of work–family conflict and to reduce the risk of job vacancy. Given its potential long-term impact on workers’ mental health and productivity, and its possible contribution to job turnover, this aspect of workers’ lives warrants active intervention by occupational health services.

## References

[1] Toyo Keizai Education . One in four parents of truant children quit or take leave: the heavy burden of home care. Article in Japanese. 2025. Accessed 28 February, 2025. https://toyokeizai.net/articles/-/852714

[2] Kanagawa AS . "I was struggling with parenting" Mother accused of murder, Ebina, three children dead. Asahi Shinbun Newspaper. p. 19 2025 4th January 2025.

[3] Ministry of Education, Culture, Sports, Science and Technology . Survey on student guidance issues, including problematic behaviors and school refusal. Article in Japanese. 2024. Accessed 28 February, 2025. https://www.mext.go.jp/a_menu/shotou/seitoshidou/1302902.htm

[4] Dee TS . Higher chronic absenteeism threatens academic recovery from the COVID-19 pandemic. Proc Natl Acad Sci USA. 2024;121(3):e2312249121. 10.1073/pnas.2312249121 38194454 PMC10801904

[5] Lester KJ, Michelson D. Perfect storm: emotionally based school avoidance in the post-COVID-19 pandemic context. BMJ Ment Health. 2024;27(1):e300944. 10.1136/bmjment-2023-300944 PMC11021743 38580437

[6] Ulaş S, Seçer İ. A systematic review of school refusal. Curr Psychol. 2024;43(21):19407-19422

[7] Bahali K, Tahiroglu AY, Avci A, Seydaoglu G. Parental psychological symptoms and familial risk factors of children and adolescents who exhibit school refusal. East Asian Arch Psychiatr. 2011;21(4):164-169 22215791

[8] Carless B, Melvin GA, Tonge BJ, Newman LK. The role of parental self-efficacy in adolescent school-refusal. J Fam Psychol. 2015;29(2):162-170. 10.1037/fam0000050 25642779

[9] McDonald B, Lester KJ, Michelson D. She didn't know how to go back': school attendance problems in the context of the COVID-19 pandemic—a multiple stakeholder qualitative study with parents and professionals. Br J Educ Psychol. 2023;93(1):386-401. 10.1111/bjep.12562 PMC10099830 36345270

[10] Mullally SL, Connolly SE. “I felt shamed and blamed”: an exploration of the parental lived experience of school distress. Front Psychiatry. 2025;16:1489316 40343095 10.3389/fpsyt.2025.1489316 PMC12058700

[11] Maynard BR, Brendel KE, Bulanda J, et al. Psychosocial interventions for school refusal with primary and secondary school students: a systematic review. Campbell Syst Rev. 2015;11(1):1-76

[12] Elliott JG, Place M. Practitioner review: school refusal: developments in conceptualisation and treatment since 2000. J Child Psychol Psychiatry. 2019;60(1):4-15. 10.1111/jcpp.12848 29197106

[13] Heyne D, King NJ, Tonge BJ, Cooper H. School refusal. Paediatric Drugs. 2001;3(10):719-732 11706923 10.2165/00128072-200103100-00002

[14] Greenhaus JH, Beutell NJ. Sources of conflict between work and family roles. Acad Manag Rev. 1985;10(1):76-88

[15] Allen TD, Herst DE, Bruck CS, Sutton M. Consequences associated with work-to-family conflict: a review and agenda for future research. J Occup Health Psychol. 2000;5(2):278-308 10784291 10.1037//1076-8998.5.2.278

[16] Oakman J, Kinsman N, Stuckey R, Graham M, Weale V. A rapid review of mental and physical health effects of working at home: how do we optimise health? BMC Public Health. 2020;20(1):1825. 10.1186/s12889-020-09875-z 33256652 PMC7703513

[17] Vyas L, Butakhieo N. The impact of working from home during COVID-19 on work and life domains: an exploratory study on Hong Kong. Policy Design Practice. 2021;4(1):59-76

[18] Watai I, Nishikido N, Murashima S. Gender difference in work-family conflict among Japanese information technology engineers with preschool children. J Occup Health. 2008;50(4):317-327 18493112 10.1539/joh.l7124

[19] Rosenthal L, Moro MR, Benoit L. Migrant parents of adolescents with school refusal: a qualitative study of parental distress and cultural barriers in access to care. Front Psychiatry. 2019;10:942 31998159 10.3389/fpsyt.2019.00942 PMC6962236

[20] Li A, Yang DD, Beauquesne A, Moro MR, Falissard B, Benoit L. Somatic symptoms in school refusal: a qualitative study among children, adolescents, and their parents during the COVID-19 pandemic. Eur Child Adolesc Psychiatry. 2024;33(7):2243-2251. 10.1007/s00787-023-02313-6 37821562

[21] Blackwell C . The impact of school challenges on parental employment among families with children on the autism spectrum. Disability Society. 2025;40(4):881-899

[22] Wright SL . Loneliness in the Workplace. Ph.D. Thesis, University of Canterbury, Christchurch, New Zealand, 2005.

[23] Sasaki N, Tsuno K, Kuroda R, et al. Workplace loneliness and job turnover: a 6-month prospective study. J Occup Health. 2025;67(1):uiaf009 39898978 10.1093/joccuh/uiaf009 PMC11879050

[24] Shimada K, Shimazu A, Geurts S, et al. Reliability and validity of the Japanese version of the Survey Work–Home Interaction–NijmeGen, the SWING (SWING-J). Community Work Fam. 2019;22(3):267-283

